# What’s Driving SNF Readmission Rates? Exploring Differences in Processes Between High and Low Performing Hospitals

**DOI:** 10.1093/geroni/igaa057.131

**Published:** 2020-12-16

**Authors:** Kirstin Manges, Roman Ayele, Marcie Lee, Chelsea Leonard, Emily Galenbeck, Robert Burke

**Affiliations:** 1 University of Pennsylvania Perelman School of Medicine, Philadelphia, Pennsylvania, United States; 2 VA Eastern Colorado Health Care System, Aurora, Colorado, United States; 3 Center for Health Equity Research and Promotion, Corporal Crescenz VA Medical Center, Philadelphia, Pennsylvania, United States

## Abstract

Despite the increasing national focus on improving post-acute care outcomes, best practices for reducing readmissions from skilled nursing facilities (SNFs) are unclear. The objective of this rapid ethnographic study was to observe processes used to prepare older patients for post-acute care in SNFs, and to explore differences between hospital-SNF pairs with high or low thirty-day readmission rates. We stratified hospitals according to readmission rates from SNF and used convenience sampling to identify two high and two low performing sites and associated SNFs (n=5). We conducted intensive multi-day observations (n=148 hours) and key informant interviews (n=30 clinicians) to describe hospital processes for discharging patients to SNF. We used thematic analysis of interviews and fieldnotes to identify differences in transitional care processes of hospitals discharging patients to SNFs. Hospitals used five major processes prior to SNF discharge that could affect care transitions for older adults: recognizing the need for post-acute care, deciding level of care, selecting SNF facility, negotiating patient fit, and coordinating care with SNF. During each stage, high-performing sites differed from low-performing sites by focusing on: 1) earlier, ongoing, systematic identification of high-risk patients; 2) discussing the decision to go to a SNF as an iterative team-based process; and 3) anticipating barriers with knowledge of transitional and SNF care processes. Identifying variations in processes used to prepare patients for SNF provides critical insight into the best-practices for transitioning patients to SNFs and areas to target for improving care of older adults.

